# 

**Published:** 1898-01-08

**Authors:** 


**Notices of Births, Deaths, and Marriages** are inserted in this column at a charge of 2s. 6d. for an announcement not exceeding four lines.

### NOTICE TO CORRESPONDENTS.

All MS., Letters, Books for Review, and other matters intended for the Editor should be addressed THE EDITOR, "THE HOSPITAL," THE HOSPITAL BUILDING, 28 & 29, SOUTHAMPTON STREET, STRAND, W.C.

The Editor cannot undertake to return rejected MS., even when accompanied by stamped directed envelope.

**Notice to Advertisers and Subscribers.**

All Advertisements, Orders for Copies of THE HOSPITAL, and Business Communications should be addressed to **THE MANAGER, (Not to The Editor),** THE HOSPITAL, The Hospital Building, 28 & 29, Southampton Street, Strand, London, W.C.

The Cost of Subscription, post free, is as follows:—Per week, 2½d. per quarter, 2s. 8d.; per half-year, 5s. 3d.; per annum, 10s. 6d. Foreign:—Per annum, 15s. 2d.; payable, in all cases, in advance.

### THE STUDENT OF MEDICINE.

### APPOINTMENTS.

G. Arnott, M.B., B.S.Durh., appointed House Surgeon to the Royal Infirmary, Newcastle-on-Tyne. J. H. Bailey, M.B.Vict., appointed District Medical Officer for Pendleton, Salford Royal Hospital. G. B. Buchanan, B.A.Camb., M.B., C.M.Glasg., appointed Dispensary Surgeon to the Western Infirmary, Glasgow. R. Davies, M.D.Edin., M.R.C.S.Eng., L.R.C.P.Lond., appointed Medical Officer to No. 3 District of the Cheltenham Union. Dr. Dixon, appointed Medical Officer of Health to the Hay Urban District Council. G. H. Edington, M.D.Glasg., M.R.C.S, L.R.C.P.Lond., appointed Extra Dispensary Surgeon to the Western Infirmary, Glasgow. H. T. Evans, M.R.C.S., L.A.H., D.P.H.Cantab., appointed Medical Officer of Health to the Bedwillty Urban District Council. Gethin-Jones, M.B., C.M., appointed Medical Officer of Health to the Porthcawl District Council. T. B. Hulke, F.R.C S.Eng., appointed Surgical Registrar to the Middlesex Hospital. W. R. Jack, M.D.Glasg., B.Sc, appointed Dispensary Physician to the Western Infirmary, Glasgow. T. Jones, M.R.C.S.Eng., L.R.C.P.Lond., L.S.A., appointed Medical Superintendent to Bootle Corporation Fever Hospital. D. Macartney, M.A.Edin., M.D.Glasg., M.B., C.M., appointed Assistant Surgeon to the Western Infirmary, Glasgow. J. R. McKinlay, L.S.A., appointed House Surgeon to the Wallasey Dispensary. G. P. Mathew, M.B.Cantab., &c., appointed Registrar to the Chelsea Hospital for Women. J. T. Neech, L.R.C.P, L.F.P.S., D.S Sc.-Vict., appointed Certifying Factory Surgeon for the Urban Districts of Atherton and Tyldesley. T. Nelson, M.D., appointed Medical Officer for the Lydney District of the Chepstow Union. R. M. Ralph, M.D., M.Ch., and L.M.R.U.I., L.A.H.Dubl., appointed Senior Assistant Medical Officer to Grove Hall Asylum, Bow, E. W. H. Rowell, M.B., B.S.Durh., appointed Senior House Physician to the Royal Infirmary, Newcastle-on-Tyne. L. A. Smith, M.B.Lond., M.R.C.S.Eng., L.R.C.P.Lond., appointed Medical Tutor to the London Hospital. S. Stephenson, M.B., F.R.C.S.E., appointed Ophthalmic Surgeon to the Evelina Hospital for Children, Southwark Bridge Road. T. Woodman, M.B., B.S.Durh., appointed Junior House Physician to the Royal Infirmary, Newcastle on-Tyne.

### PASS LIST.

### University of Cambridge.

FIRST EXAMINATION FOR MEDICAL AND SURGICAL DEGREES.
MICHAELMAS TERM, 1897.

PART I.—CHEMISTRY AND PHYSICS—The following were examined and approved: Aldridge, Armitage, Beddow, Bulley, Butterworth, Causton, Cleminson, Collingham, Colt, Cooper, Cotes Preedy, Cutler, S. Dixon, Drapes, R. S. Drew, Droop, Ellett, Ellis, H. Falk, Frankenburg, D. H. Fraser, Gardner-Medwin, Ghosh, Gibb, M. F. Grant, E. Harrison, P. Harrison, Harry, A. G. Harvey, Heath, W. R. Higgins, Horsley, Kerr, Luker, O. May, Mollison, Muil, Murphy, Neatby, Owen, Page, Plowright, R. M. Ranking, G. L. Ranking, Rees, Slade, Thresher, Ticehurst, Toppin, A. C. Warren, West, Western, Wiltshire.

PART II.—ELEMENTARY BIOLOGY.—The following were examined and approved: Akerman, Bacon, Bulley, Butterworth, J. G. Castellain, Cleminson, Colt, Crowfoot, Curl, Day, Donnell, Drapes, Ellett, Fox, D. H. Fraser, Ghosh, Gibb, Graham, M. F. Grant, Griffin, Hall, P. Hardy, Harris, P. Harrison, Hepburn, W. H. Hills, Hirst, Hudson, Irving, Leech, Leslie, Littlejohns, C. G. Martin, O. May, Molteno, Newington, Page, Panton, Patrick, Pellow, Pope, Prince, Skene, Toppin, Western, C. F. O. White, E. K. Williams, W. V. Wood, Worthington, S. L. O. Young, Young.

SECOND EXAMINATION FOR MEDICAL AND SURGICAL DEGREES.
MICHAELMAS TERM, 1897.

PART I.—PHARMACEUTICAL CHEMISTRY.—The following were examined and approved: H. Ackroyd, Barton, Beckett, Burroughes, Butler, H. J. Cardew, Carey, F. J. Child, Cooper, Crowther, Dally, Davies-Colley, Dodd, Dudley, Elliott, W. I. Evans, Freshwater, Geoghegan, Goss, Goyder, Greene, C. H. Gregory, Guthrie, Harnett, A. W. Harvey, H. G. L. Haynes, Henderson, Hocken, Holtzmann, A. C. Ingram, E. G. Kellgren, Kidd, Ledward, Leighton, Leveaux, Lloyd-Jones, Loveday, Lucas, Matthews, Murray, Muspratt, Nicholson, Norman, Paton, Pellier, Rickett, Riddle, H. J. Robinson, Rolfe, R. N. Salaman, Scarborough, C. A. St. J. Scott, Sephton, Simpson, Snell, B. Spearman, Spurrier, W. M. Strong, Tuck, Ward, L. Wigram, A. G. E. Wilcock, H. C. Williams, H. M. Wilson, Woolley, Wylie.

PART II.—HUMAN ANATOMY AND PHYSIOLOGY.—The following were examined and approved: Attlee, Badcock, Barnett, Bousfield, Bowen, Brydone, Byles, Clarke, Claxton, Crompton, Davies-Colley, Dunlop, Emerson, Foster, Gabb, Gilbart-Smith, Greene, Greig, Holmes, Home, Ingram, Kellgren, Knight, Lambert, Laycock, Leech, Lock, McBryde, Maclaren, Murray, Newman, Orton, Paterson, Paton, Perkins, Rashleigh, Sedgwick, Stirling-Hamilton, Susmann, Thomas, Ticehurst, Urwick, Weir, Woods.

